# 

**Published:** 1901-05-25

**Authors:** 


### NOTICE TO CORRESPONDENTS.

All MSS., Letters, Books for Review, and other matters intended for the Editor should be addressed The Editor, "The Hospital," The Hospital Building, 28 & 29 Southampton Street, Strand, W.C.

The Editor cannot undertake to return rejected MS., even when accompanied by stamped directed envelope.

**Notice to Advertisers and Subscribers.**

All Advertisements, Orders for Copies of The Hospital, and Business Communications should be addressed to **THE MANAGER (Not to the Editor),** The Hospital, 28 & 29 Southampton Street, Strand, London, W.C.

The Cost of Subscription, post free, is as follows:—Per week, 2½d.; per quarter, 2s. 8d.; per half-year, 5s. 3d.; per annum, 10s. 6d. Foreign:—Per annum, 15s. 2d., payable, in all cases, in advance.

**NOTICE.—Vol. XXIX. of "The Hospital" (October 1900, to March, 1901), in handsome cloth binding are now on sale at the Office of this Journal, price, 7s. 6d. Cases for binding the Numbers contained in this Volume 1s. 6d. Index to Vol. XXIX., 2½d., post free.**

**The Monthly Part for June will be ready on June 1st. Price 6d., by post 8d.**

### SCRAPS AND GLEANINGS.

THE King and Queen have consented to be patrons of the British Empire League.

223,839 patients were treated at the Manchester Hospitals during the last year.

THE usual fete of the Swindon Hospital Saturday Fund is to be held this year on Saturday, June 15th.

A CONCERT in aid of the East London Hospital for Children will be held at the Royal Albert Hall on June 8th.

THE receipts of the Bristol Orthopædic Hospital and Home for Crippled Children during the past year amounted to £1,096.

THE amount collected on Hospital Sunday this year in Reading was £176 odd, which is the largest amount ever received through this means.

THIS year's receipts for the free fund of the Coventry and Warwickshire Hospital amounted to about the same as those of last year, being £3,373 against £3,375.

THE proprietors of "Lemco" will give away next November prizes of books for collections of Lemco wrappers; particulars will be found in their advertisements.

THE Chelsea Hospital for Women has received a grant of £50 from the Grocers' Company towards the reflooring of the wards, as recommended by the Prince of Wales's Hospital Fund.

THE accommodation provided in the new Royal Victoria Hospital, Belfast, provides for 300 patients, eight resident medical and surgical officers, 76 nurses, and 32 male and female attendants and servants.

THE late Mr. Alfred Brownett has bequeathed a sum of £1,500 to the Cardiff Infirmary. Mr. Brownett was a resident in Cardiff, and started his life there first as a plasterer, and was afterwards a master builder.

DURING the past year the receipts of the Railway Mission Convalescent Home at St. Leonard's showed a falling off of £400, as compared with the previous year. The subscriptions amounted to under £3,000, of which sum £2,000 was given by railway employees.

DURING the past year the Corporation of the Sons of the Clergy, which was founded in 1655, and incorporated in 1678, has distributed a sum of £24,707 in grants to necessitous clergymen, pensions and grants to their widows, and single daughters and grants to clergymen's children.

AT the annual meeting of the Wimbledon Cottage Hospital the treasurer, in reviewing the financial position, stated that very few of the local shopkeepers contributed anything to the hospital. It was necessary to have £300 per annum in subscriptions, and even then it would be difficult to get along.

A HOLIDAY home for blind and crippled girls has been erected at Clacton-on-Sea under the auspices of the Watercress and Flower Girls' Christian Mission. Each child is sent for a fortnight or more, and they are sent down in parties of twenty throughout the warm weather. 10s. provides for each child for the fortnight, and the treasurer of the fund is F. A. Bevan, Esq., 54 Lombard Street.

### THE STUDENT OF MEDICINE.

### APPOINTMENTS.

**C. E. Abbot**, L.R.C.P.I., M.R.C.S.Eng., appointed Medical Officer for the No. 1 District of the Cheltenham Union. **A. J. Adkins**, M.D.Lond., appointed Resident Medical Officer to the Stoke-upon-Trent Union Workhouse. **A. Ashby**, M.B.Lond., appointed Medical Officer of Health for the Caversham Urban District. **E. N. Berryman,** M.R.C.S.-Eng., L.R.C.P.Lond., appointed Medical Officer for the No. 1 District and Workhouse of the Llandovery Union. **C. F. Brears,** M.D.McGill Univ., L.R.C.P., L.R.C.S.Edin., appointed District Medical Officer of the Sunderland Union. **T. B. Brierley,** M.R.C.S.Eng., appointed Certifying Factory Surgeon for the Tattenhall District of Cheshire. **C. Grainger Brown,** M.R.C.S.Eng., L.R.C.P.Edin., appointed Honorary Medical Officer to the Holloway and North Islington Dispensary. **P. E. Campbell,** M.B., C.M.Edin., appointed Medical Superintendent to the Caterham Asylum of the Metropolitan Asylums Board. **E. F. Clowes,** M.R.C.S., L.R.C.P., appointed Medical Officer to the No. 2 District of the Dussley Union, Medical Officer to the Charfield District of the Thornbury Union, Certifying Surgeon under the Factories Act to the Wotton-under-Edge District, and Surgeon to the Midland Railway Company, Charfield and Wickwor Districts. **Joseph Everett Dutton,** M.B., B.Ch.Vict., appointed to the Walter Myers Fellowship of Tropical Medicine of the Liverpool School of Tropical Medicine. **Arthur Edmunds,** M.R.C.S., L.R.C.P., appointed House-Physician to the Paddington Green Children's Hospital. **W. T. Hedley,** M.B., C.M.Edin., appointed Certifying Factory Surgeon for the Hallaton Rural District of Leicestershire. **Charles Gaskell Higginson,** M.R.C.S., L.R.C.P., L.S.A., M.A Lond., appointed Assistant Medical Superintendent at the Crooksbury Ridges Sanatorium for Consumption near Farnham, Surrey. **James Huskie,** M.B., C.M.Edin., appointed Certifying Factory Surgeon for the Crosby District of Lancashire. **A. L. Jackson,** M.D.Camb., B.C., appointed District Medical Officer of the Spalding Union. **A. Leigh,** M.R.C.S., L.R.C.P.Lond., appointed Certifying Factory Surgeon for the Malpas District of Cheshire. **R. E. Lloyd,** M.R.C.S.Eng, L.R.C.P.Lond., appointed Assistant Medical Officer to the St. Pancras Infirmary. **John McGibbon,** M.B., L.R.C.P.E., appointed a Clinical Assistant in the Observation Ward of the Edinburgh Royal Infirmary. **L. W. Crombie Macpherson,** M.B., C.M., appointed House-Surgeon to the Paddington Green Children's Hospital. **M. T. Male,** M.B.Lond., appointed Assistant Medical Officer to the St. Pancras Infirmary. **T. W. E. Moreton,** L.R.C.P.Lond., M.R.C.S.Eng., appointed Certifying Factory Surgeon for the Tarvin District of Cheshire. **H. O. Oliphant Nicholson,** M.B., C.M., appointed Clinical Assistant in the Medical Waiting Room of the Edinburgh Royal Infirmary. **V. O'Reilly,** M.B., B.Ch.R.U.I., appointed Certifying Factory Surgeon to the Civil Parish of Parbold in the Wigan Rural District. **G. E. Palmer,** M.R.C.S., appointed District Medical Officer of the Gordon Road Workhouse of the Camberwell Parish. **W. L. Pritchard,** M.B.Edin., appointed District Medical Officer of the Crickhowell Union. **Major Ronald Ross,** M.D., D.P.H., late I.M.S., appointed to the Walter Myers Chair of Tropical Medicine of the Liverpool School of Tropical Medicine. **J. W. Sandoe,** M.D.Durh., appointed District Medical Officer of the St. Thomas Union. **J. G. C. Scott,** M.B., C.M.Edin., appointed Medical Officer of Health for the Castle Donington Rural District. **W. E. Smith,** L.R.C.P., L.R.C.S.Edin., appointed District Medical Officer of the Christchurch Union. **S. H. Snell,** M.D. and B.S.Lond., M.R.C.S., D.P.H., appointed Senior Clinical Assistant to the Golden Square Throat and Ear Hospital. **J. Steed,** M.D.Edin., appointed Medical Officer to the Wyeside District of the Weobly Union. **George Turner,** M.B.Camb., D.P.H., appointed Medical Officer of Health for the Transvaal. **A. B. Vise,** L.R.C.P.-Lond., M.R.C.S.Eng., appointed District Medical Officer of the Holbeach Union.

28 & 29 SOUTHAMPTON STREET, STRAND, W.C

